# Lipomata of the Orbit

**Published:** 1885-10

**Authors:** W. L. Bullard

**Affiliations:** Columbus, Ga.


### LIPOMATA OF THE ORBIT.

BY W. L. BULLARD, M. D., COLUMBUS, GA.

On the 30th of June last Mr. W., of Eufaula, Alabama, consulted me in regard to one of his eyes, which had been troubling him for two or more years. On examination I found that the proptosis, which was very marked, was produced from a tumor situated in the orbit behind the ball, the increased growth of which with pressure from behind had caused the eye to almost protrude from its socket. There was some increase in the thickness of the upper lid, with a reduction in its muscular excursion. Patient complained of some discomfort, feeling of fullness, with slight pain resulting from the prominent globe and vascular engorgement of palpebral area. Ophthalmocscopic examination revealed a clear media, intense congestion of disc, and tortuosity and distension of retinal veins, with congestion of the choroid, and œdema of the retina, due to constant pressure from gradual enlargement of tumor, which caused an interference with the intra-ocular circulation, resulting in partial suspension in the function of the eye; in fact, vision was very defective, only $\frac{xv}{200.}$ After detailing the consequences which sometimes arise from such an operation, the patient, after being told no benefit could be given save extirpation of tumor, readily consented to an operation. Dr. Blanchard, of this city, kindly assisted me, and after anesthetizing patient, the operation was made. Due to the excessive varicose condition of vessels in the palpebral fold, the usual line of incision was deviated from. Instead of making a conjunctival incision with canthoplasty, etc., the incision was carried through the skin of the upper lid, horizontally and slightly curved so as to correspond to the natural folding of the skin, and thus avoid any disfigurement. After making an incision about two inches long, and cutting down to the growth, then with a pair of scissors curved on the flat extirpation was accomplished with but little difficulty. The growth was about the size of a walnut, and after its enucleation the ball readily fell back into its socket. Hemorrhage was easily checked by pressure, and the edges of the wound brought together and retained in apposition with several sutures. The wound healed by first intention, and on the third day patient was dismissed as cured, save slight ptosis of upper lid from division of the levator palpebra. Vision rapidly improved and after the expiration of a week was almost normal. The patient left for home, advised if ptosis continued to present himself in the course of six or eight weeks, so that an operation might be performed on the lid to correct its defect. Up to this writing have heard nothing more from the case. (I might mention that on the day the case in question presented itself, I had another patient to consult me who had exophthalmus of the same—right—eye, though brought about from a different cause—distention of the frontal sinuses. I advised an operation, but up to this time patient has not consented to have it done.) In reporting this case I am prompted to do so on account of its infrequency. With no little experience in the eye and ear hospitals, both in America and Europe, I must say that the number of cases like this, seen by the writer, are quite limited.

